# Eriodictyol attenuates dextran sodium sulphate-induced colitis in mice by regulating the sonic hedgehog signalling pathway

**DOI:** 10.1080/13880209.2021.1948066

**Published:** 2021-08-04

**Authors:** Ru Wang, Lei Shen, Huimin Li, Hao Peng

**Affiliations:** a Department of Gastroenterology, Renmin Hospital of Wuhan University, Hubei Key Laboratory of Digestive System Diseases, Wuhan, P.R. China; b Department of Gastroenterology, Renmin Hospital of Wuhan University, Wuhan, P.R. China

**Keywords:** IBD, inflammation, oxidative stress, epithelial barrier destruction

## Abstract

**Context:**

Eriodictyol (EDT) is a flavonoid with strong anti-inflammatory, anti-apoptotic, and antioxidant properties.

**Objective:**

To investigate the protective effect and mechanism of EDT in ulcerative colitis (UC).

**Materials and methods:**

UC model was induced by 3% dextran sulphate sodium (DSS) solution for 7 days, meanwhile, EDT and Smoothened (Smo) inhibitor cyclopamine (Cyc) were intraperitoneally injected. In the first experiment, C57BL/6 mice divided into blank control, DSS, DSS + EDT (20 or 40 mg/kg) groups. In second experiment, added Cyc (5 mg/kg) and EDT + Cyc groups. All mice were sacrificed on day 8. Disease activity index (DAI), colon length and colon histology as well as MDA levels, SOD, and GSH-Px activities were measured. The expression of Sonic hedgehog (Shh), Patched, Smo, glioblastoma-1, zonula occludens-1 (ZO-1), occludin, cleaved caspase 3, Bax and Bcl-2 in colon was detected using RT-PCR and Western blotting.

**Results:**

After EDT treatment, compared with the DSS group, DAI (2.33 ± 0.516 vs. 3.67 ± 0.516), colon shortening (5.27 ± 0.476 vs. 4.53 ± 0.528 cm) and histological score (6.67 ± 1.211 vs. 12 ± 1.265) was significantly decreased. EDT also reduced inflammation, oxidative stress and apoptosis in colon. Additionally, EDT increased the expression of the tight junction proteins ZO-1 (35%) and occludin (66.3%). Mechanistically, EDT upregulated the Shh signalling pathway. However, Cyc-mediated inhibition of the Shh pathway partially abolished the effects of EDT.

**Discussion and Conclusions:**

These results indicate EDT attenuates DSS-induced colitis by activating the Shh pathway. Further clinical trials are needed to demonstrate its efficacy on UC.

## Introduction

Ulcerative colitis (UC) is a major subtype of chronic, relapsing, non-specific inflammatory bowel disease (IBD). It is characterized by recurrent inflammation, usually involving the rectum and colon, followed by prolonged remission. Although the pathogenesis of UC is not clear, it is generally thought to be related to immune dysfunction, genetic susceptibility, intestinal flora imbalance, environmental factors or other factors (Kaser et al. 2010). Oxidative stress, the inflammatory response, and epithelial barrier destruction are considered important intracellular events that contribute to UC. Thus, finding drugs for these targets may be a promising approach for UC.

Hedgehog (Hh) signalling is a critical pathway that regulates patterning events and the morphogenesis of the gut in early embryos (Kolterud et al. 2009). Three ligands mediate the Hh signalling pathways: Sonic Hedgehog (Shh), Indian Hedgehog (Ihh) and Desert Hedgehog (Dhh). Shh is one of the most relevant Hh ligands in the human intestine and the Shh signalling pathway is the most frequently studied among the Hh signalling pathways. Shh signalling regulates inflammatory responses, oxidative stress, epithelial cell apoptosis and tight junctions (TJs) in the gastrointestinal tract (GI) (Zavros 2008; Xiao et al. 2010; Chen et al. 2017). The major components of the Shh signalling pathway include the Shh ligand, the Patched (Ptc) and Smoothened (Smo) receptors, and the transcription factor Glioblastoma-1 (Gli1) (Varjosalo et al. 2006). The Shh ligand interacts with the Ptc receptor, inducing its activation, and activated Ptc interacts with the second membrane receptor Smo, transmitting downstream signals and triggering the nuclear translocation of the transcription factor Gli1 (Briscoe and Therond 2013). When acute colitis was induced by dextran sodium sulphate (DSS), Gli1^+/lacZ^ mice that express only half the normal amount of the Gli1 protein developed more severe intestinal inflammation than wild-type mice, and IL-23/IL-17 signalling was significantly increased (Lees et al. 2008). In a bi-transgenic mouse model of chronic Hh inhibition, chronic inhibition of Hh signalling in adult animals led to spontaneous intestinal inflammation (Zacharias et al. 2010). Additionally, compared with wild-type mice, mice in which the activity of the Hh pathway was reduced by pharmacological inhibition of Smo and Smo ablation exhibited an increased severity of DSS-induced colitis. Conversely, an increase in the activity of the Hh pathway decreased DSS-induced colitis (Lee et al. 2016).

Numerous studies have shown that flavonoids have therapeutic potential not only in colitis mice but also in human UC (Biedermann et al. 2013; Li et al. 2016; Salaritabar et al. 2017). Eriodictyol (EDT), a flavonoid commonly found in citrus fruits and vegetables, has been reported to have anti-inflammatory, anti-apoptotic, and antioxidant properties. EDT has been reported to significantly inhibit NO production in LPS-treated RAW 264.7 murine macrophages (Dai et al. 2008). EDT also prevents early retinal and plasma abnormalities in streptozotocin-induced diabetic rats (Bucolo et al. 2012). According to another study, EDT protects against As_2_O_3_-induced liver injury by increasing the activity of antioxidant enzymes and inhibiting the production of proinflammatory factors (Xie et al. 2017). In addition, EDT reduces the levels of Bax and cleaved caspase 3, and increases Bcl-2 levels to alleviate myocardial apoptosis in myocardial ischaemia-reperfusion model (Li et al. 2018). However, the effects and exact mechanism of EDT on colitis remain unclear. Therefore, this study was carried out in two parts. First, we studied the effects of EDT on inflammation, oxidative stress, and epithelial barrier disruption in mice with colitis. Second, we confirmed whether the effect of EDT is related to Shh pathway activation.

## Materials and methods

### Animals

Male C57BL/6 mice (age, 6–8 weeks old; weight, 22–24 g) were purchased from Beijing Vital River Laboratory Animal Technology Co., Ltd. (Beijing, China). All mice were raised in the specific pathogen-free animal centre in Renmin Hospital at Wuhan University under controlled laboratory conditions (21 ± 2 °C, 0 ± 5% humidity, and a 12-h light/dark cycle). The mice had access to a standard laboratory diet and sterile water *ad libitum* during the experimental period. Before the study began, the animals were acclimated for one week at the Experimental Animal Laboratory. Research involving animals was approved by the Laboratory Animal Welfare & Ethics Committee (IACUC) of Renmin Hospital of Wuhan University, Wuhan, China (No. WDRM20180413). All experimental protocols were conducted in accordance with the guidelines of the Animal Care and Use Committee of Renmin Hospital of Wuhan University, Wuhan, China.

### Reagents

EDT (purity > 98%) was purchased from Shanghai Yuanye Biotechnology Co., Ltd. (Shanghai, China). Dextran sulphate sodium (DSS; MW 36,000–50,000 Da) was obtained from MP Biomedicals (Aurora, OH, USA). Cyclopamine (Cyc; purity ≥ 99%) was purchased from Aladdin Industrial Corporation (Shanghai, China). Superoxide dismutase (SOD), glutathione peroxidase (GSH-Px) and malondialdehyde (MDA) reagent kits were purchased from the Nanjing Jiancheng Bioengineering Institute (Nanjing, China). The anti-Shh antibody was purchased from Santa Cruz Biotechnology Inc. (CA, USA). Antibodies against Bcl-2, Bax, Gli1, Ptc, and Smo were purchased from Abcam (Cambridge, UK), while antibodies against occludin, zonula occluden-1 (ZO-1), and cleaved caspase 3 were purchased from Affinity (Beijing, China). The β-actin antibody was purchased from Boster Biological Technology Co. (Wuhan, China).

### Experimental design

**Figure 1.**
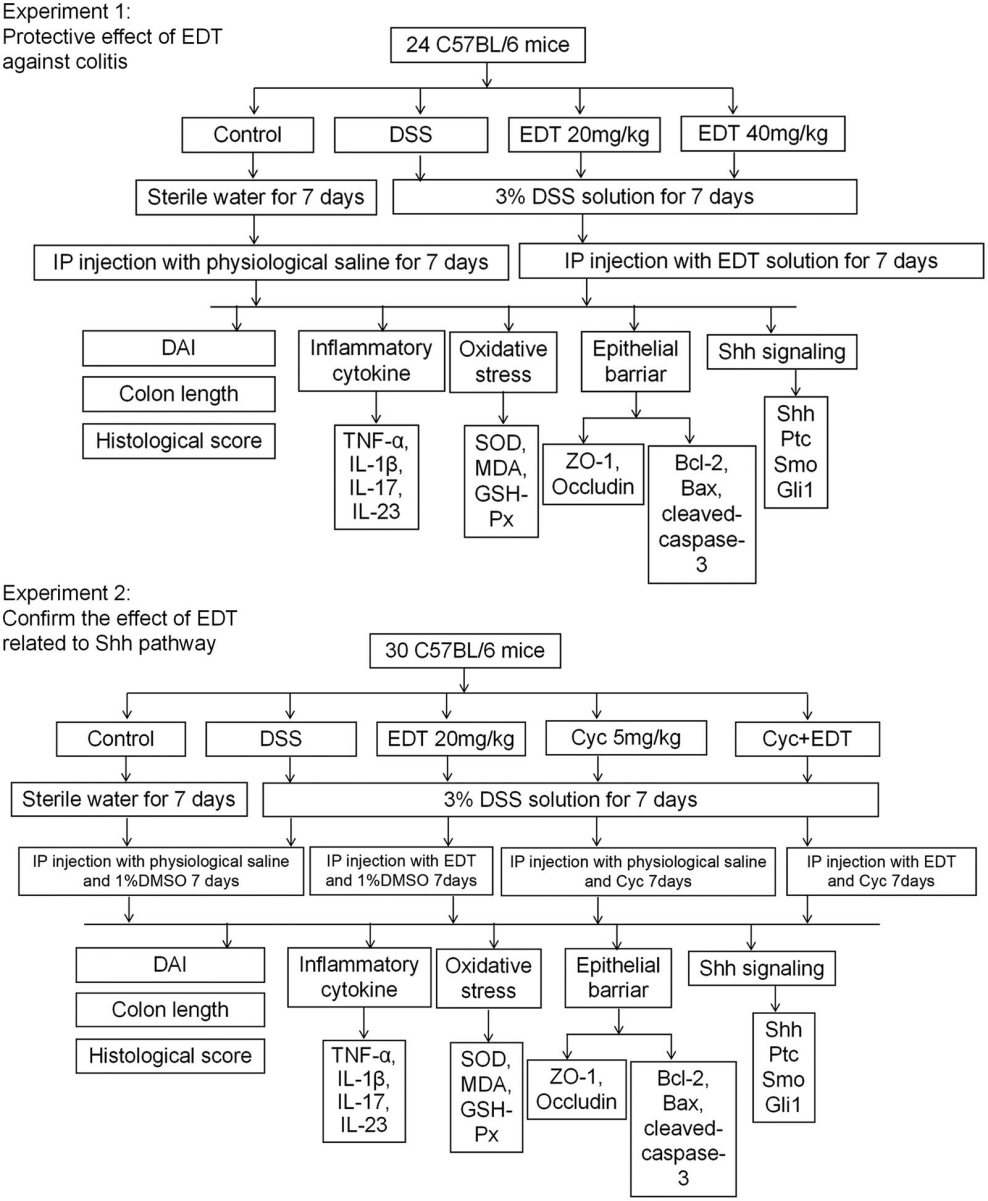
Experiment design. DSS: dextran sulphate sodium; EDT: eriodictyol; Cyc: cyclopamine.

To assess the protective effects of EDT against colitis and to confirm the role of EDT in activating the Shh signalling pathway during colitis protection, we designed two experiments, as shown in Figure 1. The colitis model was induced in C57BL/6 mice with the administration of a 3% DSS solution for 7 days. For study 1, 24 mice were randomly divided into four groups (*n* = 6/group): (1) control group; (2) DSS model group; and (3)–(4) DSS + EDT groups (20 or 40 mg/kg) (Li et al. 2016). The mice in the control and DSS model groups were intraperitoneally injected with physiological saline daily for 7 days, while the mice in the DSS + EDT (20 or 40 mg/kg) groups were intraperitoneally injected with EDT dissolved in physiological saline daily for 7 days.

For study 2, 30 mice were randomly divided into five groups (*n* = 6/group): (1) control condition; (2) DSS treatment; (3) DSS plus EDT treatment (20 mg/kg); (4) DSS plus Cyc treatment (5 mg/kg) (Zhou et al. 2014); (5) DSS plus EDT treatment (20 mg/kg) and Cyc treatment (5 mg/kg). Cyc (5 mg/kg) was diluted in 1% dimethyl sulfoxide (DMSO) for the intraperitoneal injection.

All mice were sacrificed on day 8, and the entire colon was removed. After the length of each colon was measured, the colon was washed with cold PBS. The distal colon tissue was excised and fixed with 4% paraformaldehyde for the histopathological analysis. The residue was frozen in liquid nitrogen and stored in a −80 °C freezer for further analysis.

### Disease activity index calculation

The body weight, stool consistency, and gross bleeding scores of all mice were recorded during the experimental period. The disease activity index (DAI) score was calculated as the sum of the weight loss, stool consistency and faecal blood content according to the following criteria (Ito et al. 2006): body weight loss (0: normal, 1: 1–5% weight loss, 2: 6–10% weight loss, 3: 11–20% weight loss, 4: more than 20% weight loss), change in stool blood (0: negative, 2: hemoccult positive, 4: gross bleeding), and stool consistency (0: normal, 2: loose stool, 4: diarrhoea).

### Histopathological analysis

Distal colon tissues fixed with 4% paraformaldehyde were embedded in paraffin, stained with haematoxylin and eosin (H&E) and then observed with an optical microscope. The histological score was determined according to a previous report (Zhang et al. 2014): % tissue damage (0: no tissue damage, 1: 1–25% damaged tissue, 2: 26–50% damaged tissue, 3: 51–75% damaged tissue, 4: 76–100% damaged tissue), extent of tissue damage (0: no tissue damage, 1: mucosal damage, 2: mucosal and submucosal damage, 3: damage beyond the submucosa), degree of inflammation (0: no inflammation, 1: slight inflammation, 2: moderate inflammation, 3: severe inflammation), extent of crypt damage (0: no crypt damage, 1: basal 1/3 showed damage, 2: basal 2/3 showed damage, 3: only the surface epithelium was intact, 4: the entire crypt and epithelium were lost).

### Determination of oxidative stress and antioxidant enzymes

The content of MDA and the activity levels of SOD and GSH-Px in colon tissue homogenates were determined using a commercial kit (Nanjing Jiancheng Bioengineering Institute, Nanjing, China) by spectrophotometry according to the manufacturer's protocols.

### Real-time PCR

According to the manufacturer's protocol, total RNA was extracted from colon samples using TRIzol reagent (Ambion, Austin, TX, USA). The cDNA was reverse transcribed from the extracted RNA using Reverse Transcriptase (Thermo Scientific, USA). Real-time PCR was conducted with the SYBR Green probe, and ABI 7500. β-Actin was used as a reference gene. The sequences of all primers used for RT-PCR are shown in Table 1.

**Table 1.** Primer sequences for RT-PCR.

Name	Primer	Sequence
β-actin	Forward	5′-CACGATGGAGGGGCCGGACTCATC-3′
	Reverse	5′-TAAAGACCTCTATGCCAACACAGT-3′
TNF-α	Forward	5′-ACCCTCACACTCACAAACCA-3′
	Reverse	5′-GGCAGAGAGGAGGTTGACTT-3′
IL-1β	Forward	5′-TCAGGCAGGCAGTATCACTC-3′
	Reverse	5′-AGCTCATATGGGTCCGACAG-3′
IL-17	Forward	5′-AGACTACCTCAACCGTTCCAC-3′
	Reverse	5′-CAGCTTTCCCTCCGCATT-3′
IL-23	Forward	5′-AGCTCTCTCGGAATCTCTGC-3′
	Reverse	5′-ACTGGCTGTTGTCCTTGAGT-3′
Shh	Forward	5′-GATGAGGAAAACACGGGAGC-3′
	Reverse	5′-CTGCTCGACCCTCATAGTGT-3′
Ptc	Forward	5′-CCGCATTGATCCCTATCCCT-3′
	Reverse	5′-GGGTGTTACTGTGAGGCTCT-3′
Smo	Forward	5′-GGACATGCACAGCTACATCG-3′
	Reverse	5′-CTCGGCAAACAATCTCTCGG-3′
Gli1	Forward	5′-TCTGTGATGGGCAATGGTCT-3′
	Reverse	5′-TCTGGGGTGGGATCAGGATA-3′

### Western blot analysis

According to the manufacturer's protocol (Thermo Fisher Scientific Inc., USA), nuclear and cytosolic proteins were extracted from the colon tissues. The protein concentration was quantified by the BCA method. Equal amounts (40 μg) of protein were subjected to SDS-PAGE and then transferred to polyvinylidene difluoride (PVDF) membranes (Millipore, USA). The membranes were blocked with TBST containing 5% skim milk for 2 h at room temperature and incubated overnight at 4 °C with primary antibodies against ZO-1 (1:1000 dilution), occludin (1:1000 dilution), Shh (1:1000 dilution), Smo (1:1000 dilution), Ptc (1:500 dilution), Gli1 (1:500 dilution), Bcl-2 (1:1000 dilution), Bax (1:1000 dilution), cleaved caspase 3 (1:1000 dilution), and the internal reference β-actin (1:2000 dilution). The PVDF membranes were fully washed 5–6 times with TBST and then incubated with the appropriate HRP-conjugated secondary antibodies (1:50000 dilution) for 2 h at room temperature. Immunoreactive bands were detected by enhanced chemiluminescence and quantified using ImageJ software (NIH, Bethesda, MD, USA).

### Statistical analyses

The normal distribution of quantitative data was tested with the Kolmogorov–Smirnov test. The data were normally distributed, and the statistical significance of differences among multiple groups was determined with one-way ANOVA followed by Tukey’s *post hoc* test. All data were analysed by SPSS 22.0 software and expressed as the mean ± SD, as indicated in the figure legends. A value of *p* < 0.05 was considered statistically significant.

## Results

### Influence of EDT on body weight, DAI score, colon length and histological damage

**Figure 2.**
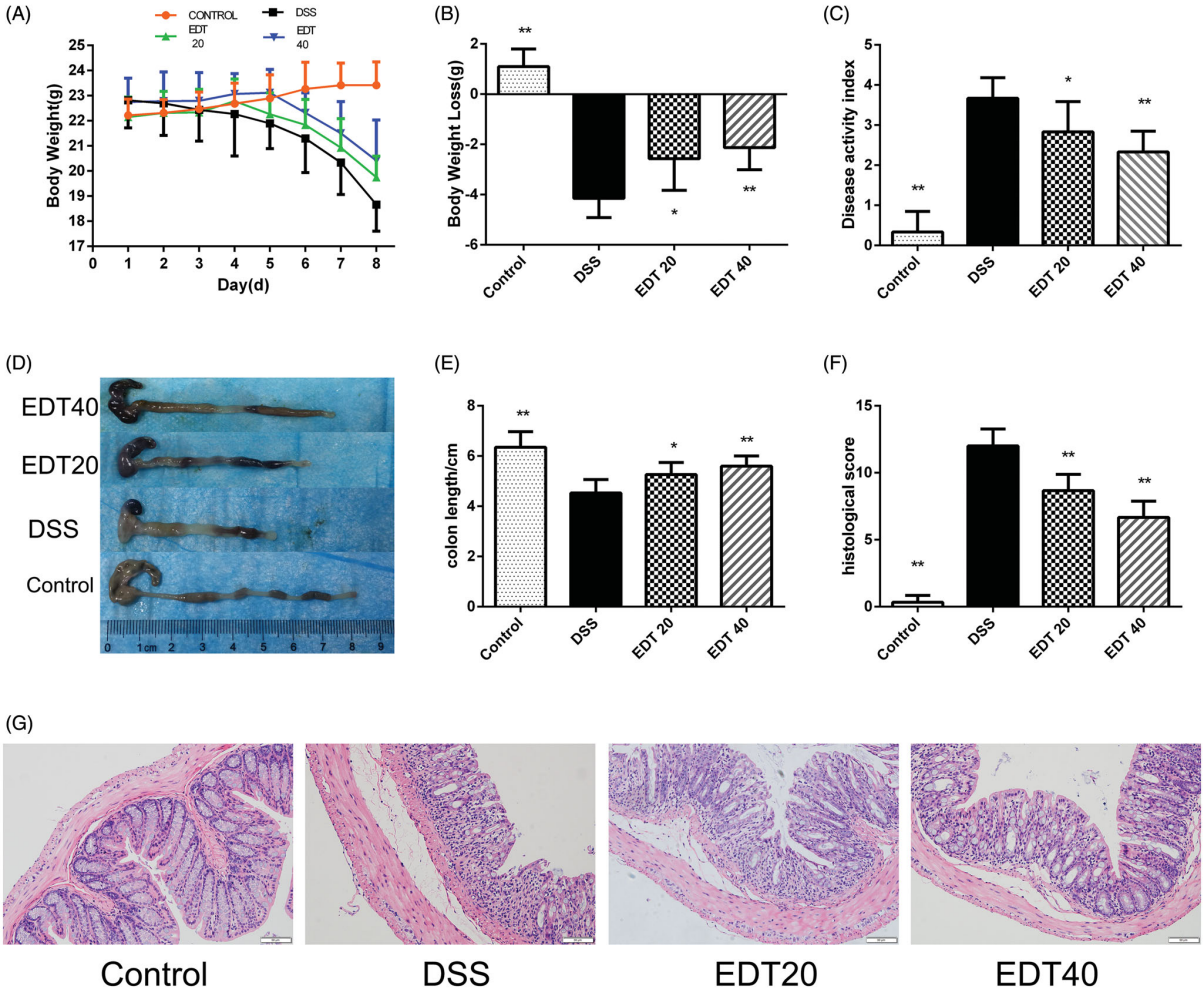
Effect of EDT on disease activity and histological damage in mice with DSS-induced colitis. (A) Body weights recorded in the experimental period. (B) Body weight loss was reported in grams. (C) The DAI score. (D) Representative macroscopic images of colon tissues from mice in each group. (E) Colon length. (F) The histopathology score. (G) Representative images of the colonic sections stained with H&E (magnification × 200, scale bars = 50 µm). The results are presented as means ± S.D. (*n* = 6). DSS: dextran sulphate sodium; EDT: eriodictyol; *p < 0.05 and **p < 0.01 compared with the DSS model group.

The DAI score was determined by body weight loss, stool consistency and the presence of blood in the stool. We found that during the experiment, all groups of mice, except for the control group, lost weight. The mice in the DSS group had significantly more weight loss than the mice in the control group (*p* < 0.01), and the weight loss of the EDT treatment group was less than that of the DSS group (*p* < 0.01 or *p* < 0.05; Figure 2(A–B). The DAI score of the mice in the DSS group was higher than that of the mice in the control group (*p* < 0.01), and a lower DAI score was observed in the EDT group than in the DSS group (*p* < 0.01 or *p* < 0.05; Figure 2(C)). As shown in Figure 2(D–E), the mice in the DSS group had shortened colons, and treatment with a low dose (20 mg/kg) or a high dose (40 mg/kg) of EDT significantly inhibited colon shortening (*p* < 0.05 or *p* < 0.01). Moreover, EDT treatment improved colonic histological damage. In the control group, the colon tissues showed a normal structure, without any damage. The colons of the DSS model group had structural abnormalities, such as mucosal ulceration, crypt degeneration, and inflammatory cell infiltration. The administration of EDT ameliorated these histological changes (Figure 2(G)). The histological score was increased in the model group compared with the control group (*p* < 0.01) and markedly decreased by EDT treatment (*p* < 0.01; Figure 2(F)).

### EDT suppresses the expression of pro-inflammatory cytokines

**Figure 3.**
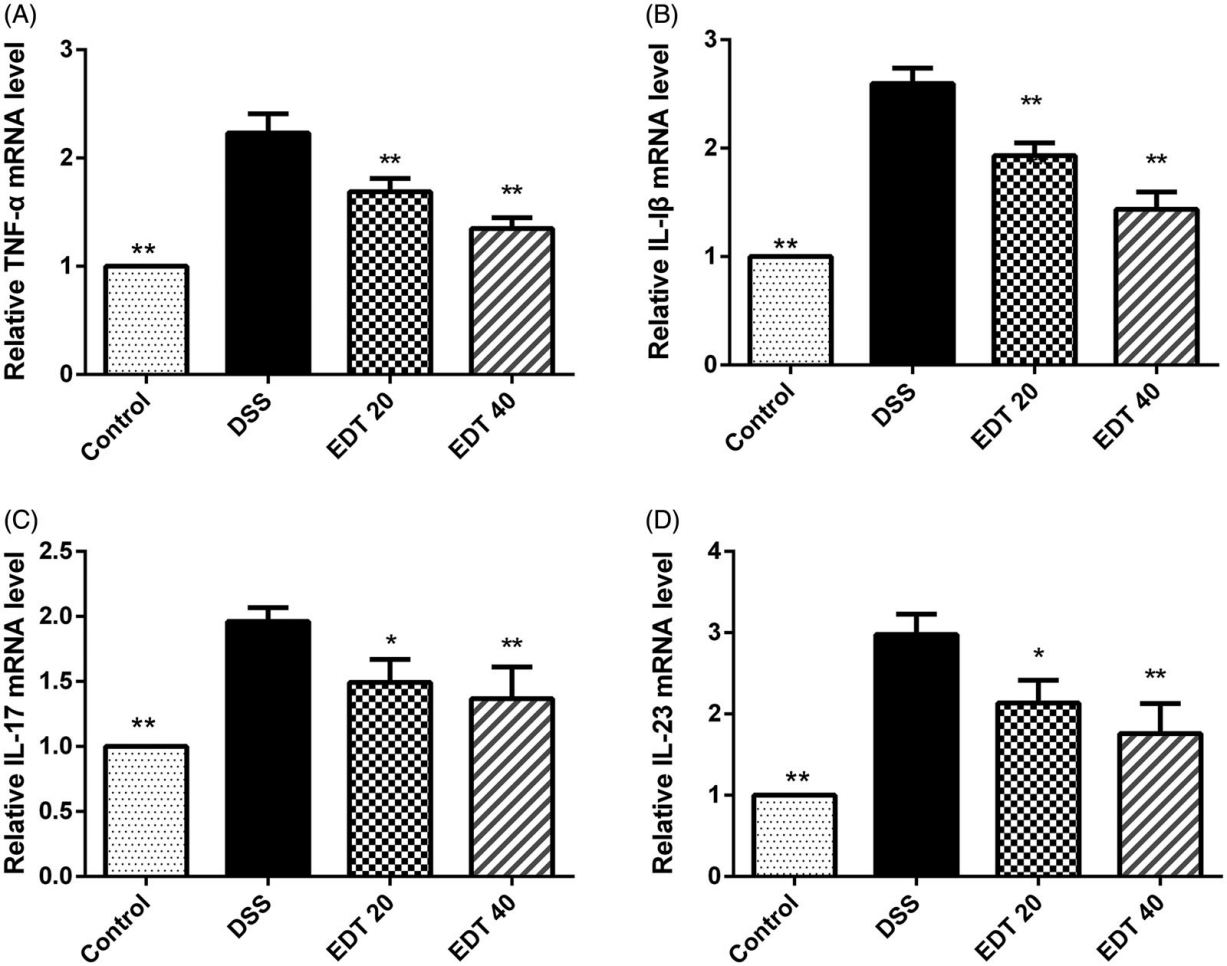
The EDT treatment suppresses the increased expression of pro-inflammatory cytokines in mice with DSS-induced colitis. The effect of EDT on TNF-α (A), IL-1β (B), IL-17 (C), and IL-23 (D) levels in colon tissues was measured using RT-PCR. All data are presented as means ± S.D. (*n* = 6). DSS: dextran sulphate sodium; EDT: eriodictyol; *p < 0.05 and **p < 0.01 compared with the DSS model group.

To investigate the effect of EDT on the expression of pro-inflammatory cytokines in DSS-induced colitis, the expression levels of TNF-α, IL-1β, IL-17, and IL-23 in colon tissues were measured by real-time PCR. As shown in Figure 3(A–D), the mRNA levels of TNF-α, IL-1β, IL-17, and IL-23 were dramatically increased in the DSS model group compared with the healthy control group, and the elevated expression levels of these cytokines were effectively suppressed by EDT treatment (*p* < 0.05 or *p* < 0.01).

### The protective effect of EDT against barrier disruption

**Figure 4.**
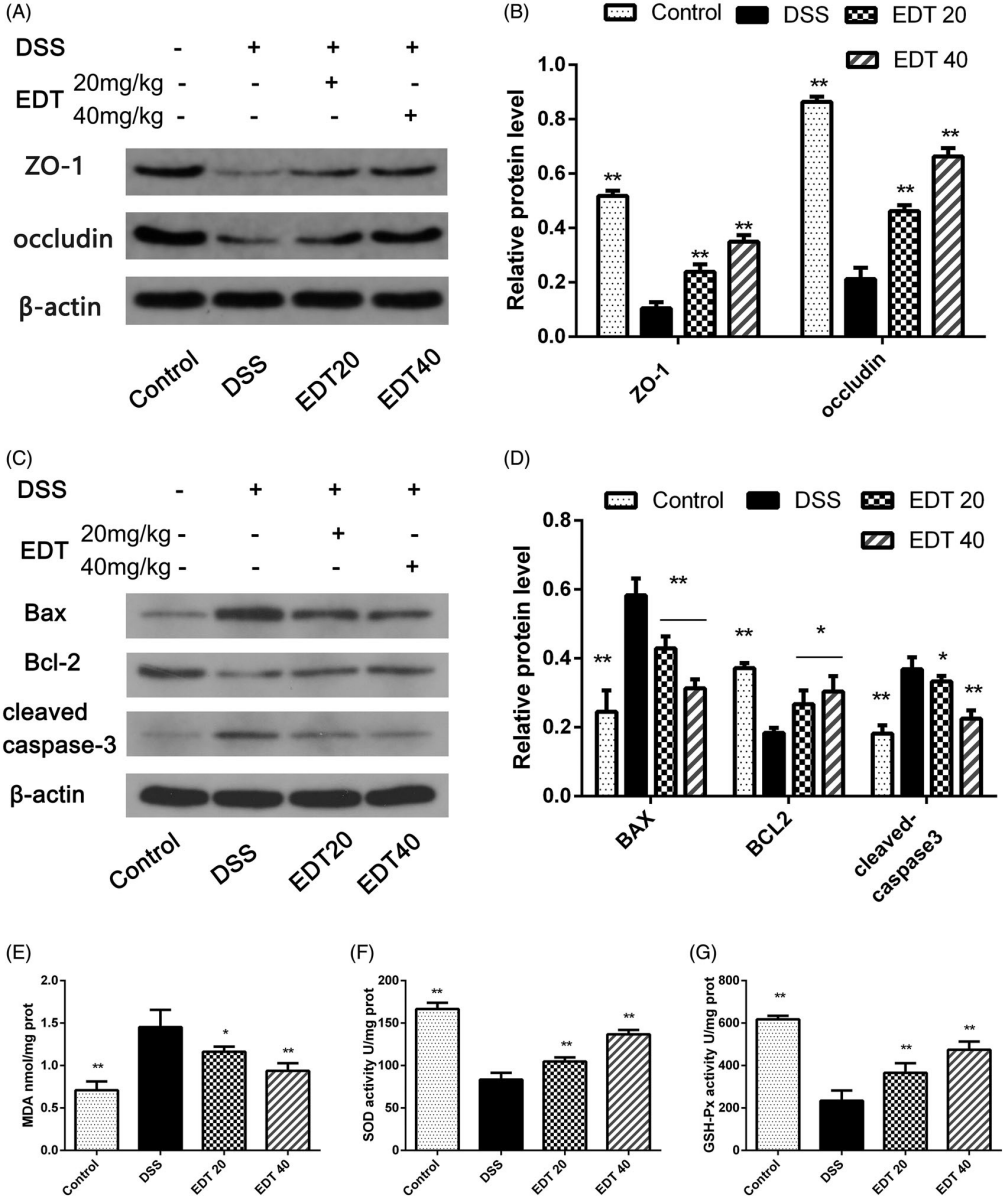
EDT protects against barrier disruption and improves oxidative stress in the colon. (A-B) Levels of the TJ proteins ZO-1 and occludin were assessed using Western blot analyses. (C-D) Levels of Bax, cleaved caspase 3, and Bcl-2 were measured using Western blot analyses. (E) The MDA content, (F) SOD activity, (G) GSH-Px activity were determined in colon tissues. All data are presented as means ± S.D. (*n* = 6). DSS:: dextran sulphate sodium; EDT: eriodictyol; *p < 0.05, **p < 0.01 compared with the DSS model group.

The loss of TJ proteins (such as ZO-1 and occludin) and the apoptosis of intestinal epithelial cells are the main factors that contribute to barrier disruption. In this study, we examined the expression levels of TJ proteins and the apoptosis-related factors Bax, Bcl-2 and cleaved caspase 3 in mouse colon tissues by Western blot analysis. As shown in Figure 4(A–D), the protein levels of ZO-1 and occludin were significantly decreased in the DSS group compared with the control group (*p* < 0.01) and significantly increased by EDT treatment (*p* < 0.01). The levels of the pro-apoptotic proteins cleaved caspase 3 and Bax in the colon were markedly increased in the DSS group compared to the control group (*p* < 0.01), and this increase was dramatically reversed by EDT treatment (*p* < 0.05 or *p* < 0.01). The level of the anti-apoptotic factor Bcl-2 was significantly decreased in the DSS group compared with the control group (*p* < 0.01), and the DSS-induced level of Bcl-2 was significantly increased by EDT treatment (*p* < 0.05). Thus, EDT treatment protects against barrier damage by attenuating TJ protein loss and attenuating apoptosis in mice with colitis.

### EDT improves oxidative stress

As shown in Figure 4(E–G), compared to the control condition, DSS treatment significantly upregulated the MDA content in the colons of mice (*p* < 0.01); compared with DSS treatment, EDT treatment obviously downregulated the MDA content (*p* < 0.05 or *p* < 0.01). The enzymatic activity of SOD and GSH-Px was significantly downregulated in the DSS group compared with the control group (*p* < 0.01). Interestingly, EDT treatment upregulated the enzymatic activity of SOD and GSH-Px (*p* < 0.01).

### EDT increases the activation of the shh pathway

**Figure 5.**
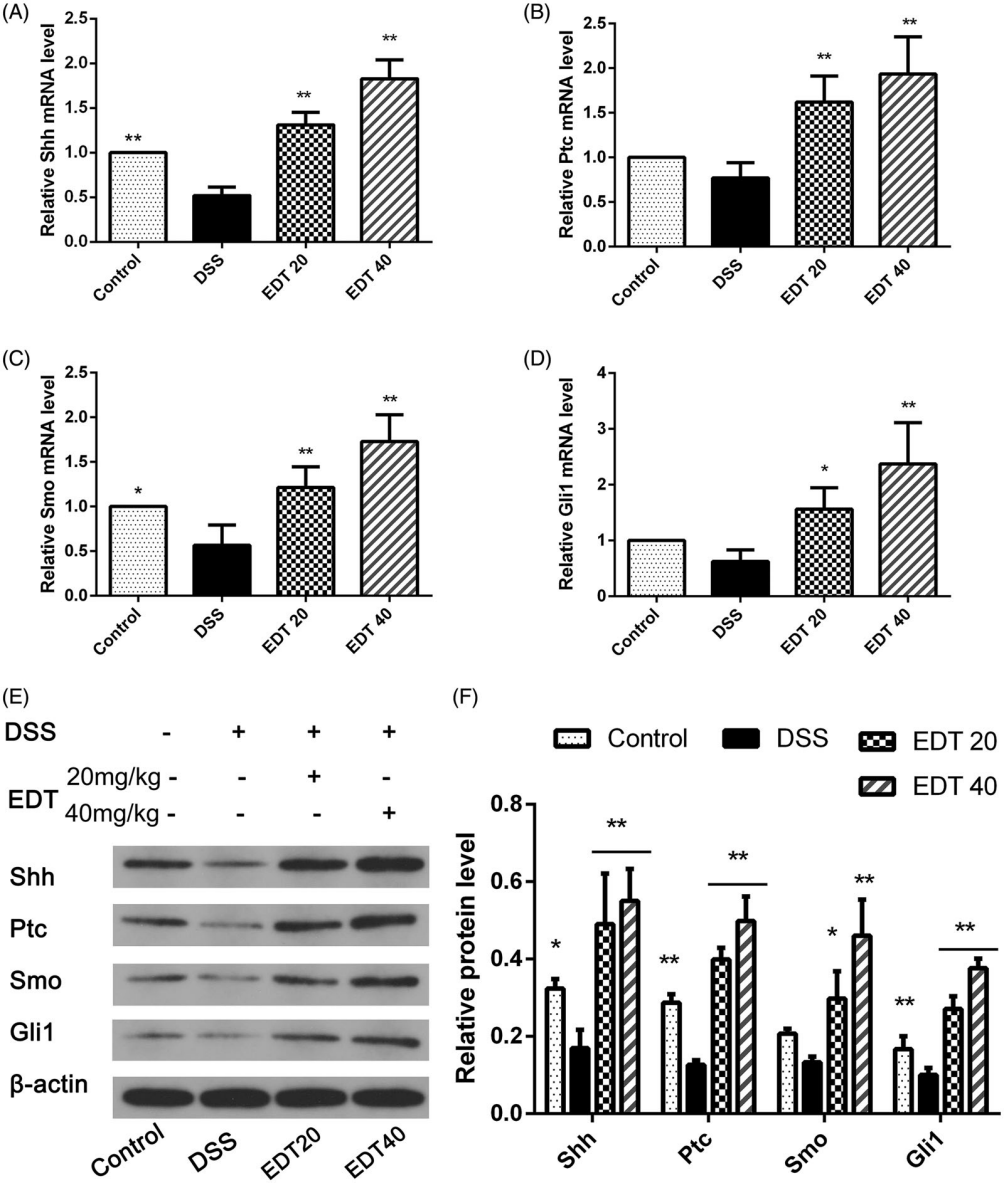
EDT increases the activation of the Shh pathway in mice with DSS-induced colitis. (A-D) Levels of the Ptc, Shh, Smo, and Gli1 mRNAs were measured using RT-PCR. (E-F) Levels of the Shh, Ptc, Smo, and Gli1 proteins were determined using Western blot analysis. All data are presented as means ± S.D. (*n* = 6). DSS: dextran sulphate sodium; EDT: eriodictyol; *p < 0.05 and **p < 0.01 compared with the DSS model group.

To further explore the mechanism of EDT protection, we determined the protein and mRNA levels of Shh pathway components in colon tissue from DSS-induced colitis mice. As shown in Figure 5, RT-PCR and Western blot analyses showed that expression of Shh, Ptc, Smo and Gli1 was reduced in the DSS group compared with the control group. After EDT treatment and that expression of Shh, Ptc, Smo and Gli1 was obviously increased. Moreover, there was a significant difference between the EDT treatment groups (20 and 40 mg/kg) and the DSS group (*p* < 0.05 or *p* < 0.01).

### Cyc aggravates DSS-induced colitis

**Figure 6.**
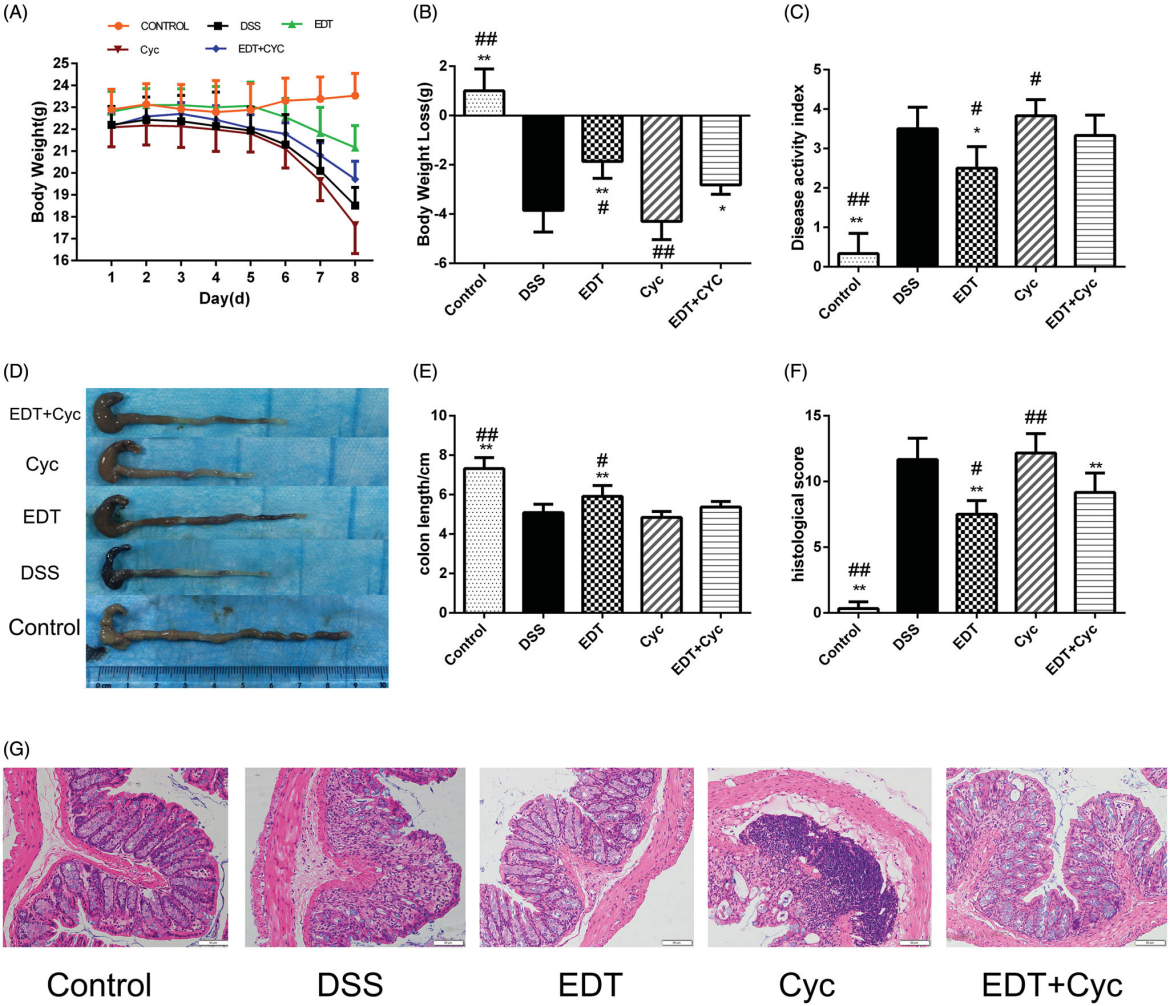
Cyc aggravates DSS-induced colitis. (A) Body weights recorded in the experimental period. (B) Body weight loss was reported in grams. (C) The DAI score. (D) Representative macroscopic images of colon tissues. (E) Colon length. (F) The histopathology score. (G) Representative images of the colonic sections stained with H&E (magnification × 200, scale bars = 50 µm). Data are presented as means ± S.D. (*n* = 6). DSS: dextran sulphate sodium; EDT: eriodictyol; Cyc: cyclopamine; *p < 0.05 and **p < 0.01 compared with the DSS group; #*p* < 0.05 and ##*p* < 0.01 compared with the EDT + Cyc group.

To further confirm whether Shh pathway activation is involved in the protective effect of EDT on DSS-induced colitis, we used Cyc, a specific Smo receptor inhibitor, to inhibit Shh pathway activation and subsequently evaluated the effect of Cyc treatment on weight loss, DAI score, histological score and colon length. The results showed that blocking the Shh pathway by treatment with the Shh inhibitor Cyc exacerbated weight loss (Figure 6(A–B)), increased the DAI score (Figure 6(C)), aggravated histological signs of colitis and increased the histological score (Figure 6(F–G)). Furthermore, the length of the colon significantly decreased (Figure 6(D–E)). Thus, treatment with the Shh pathway inhibitor Cyc attenuates the protective effect of EDT administration and aggravates the severity of colitis.

### Cyc reverses the effect of EDT on the inflammatory response

**Figure 7.**
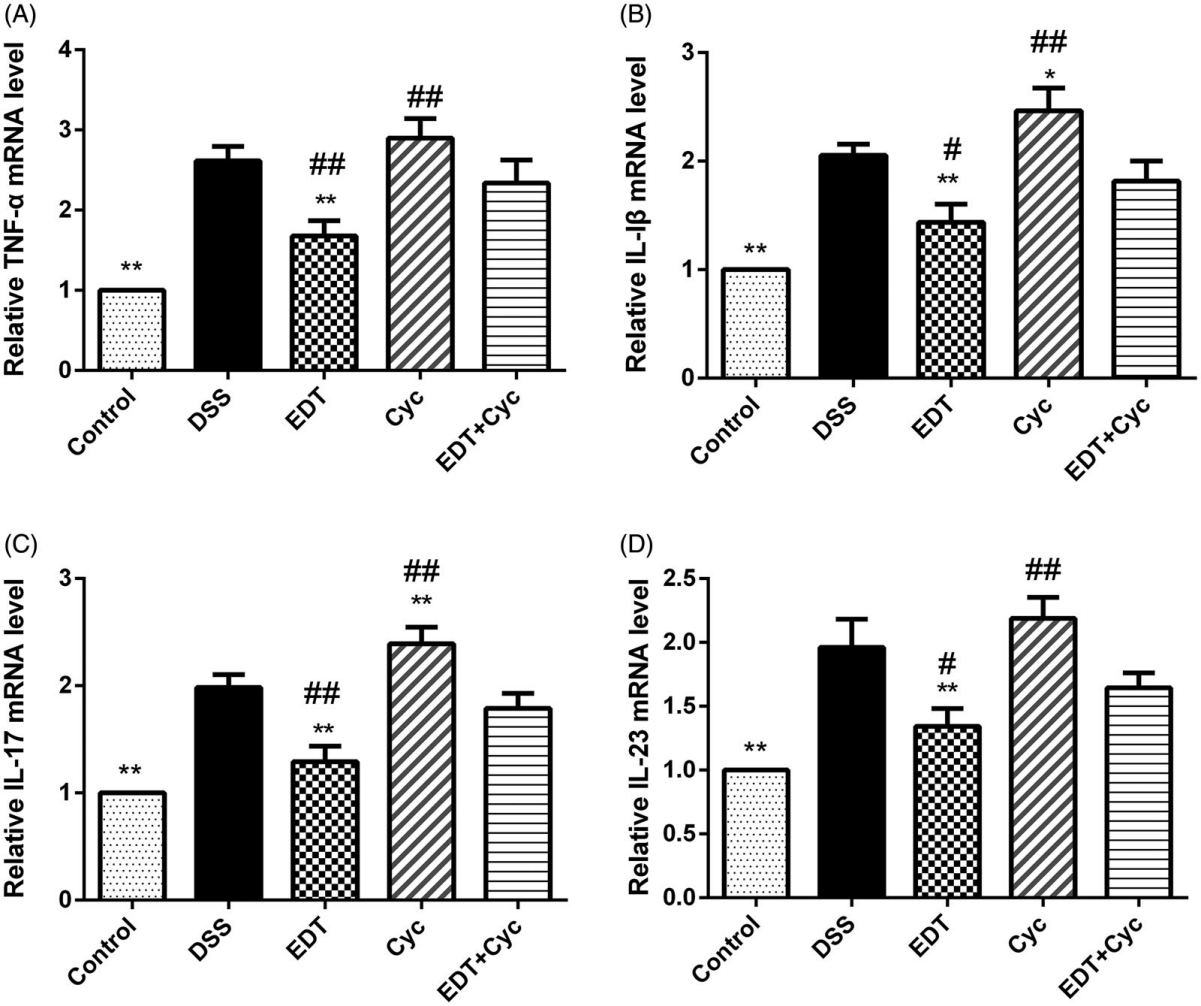
Cyc reverses the effect of EDT on the inflammatory response in mice with DSS-induced colitis. The levels of (A) TNF-α, (B) IL-1β, (C) IL-17, and (D) IL-23 in colon tissues were measured using RT-PCR. Data are presented as means ± S.D. (*n* = 6). DSS: dextran sulphate sodium; EDT: eriodictyol; Cyc: cyclopamine; *p < 0.05 and **p < 0.01 compared with the DSS group; #*p* < 0.05 and ##*p* < 0.01 compared with the EDT + Cyc group.

mRNA levels of IL-1β, IL-17, IL-23 and TNF-α were significantly increased in the DSS model group compared with the healthy control group, and levels of these inflammatory cytokines were further increased in the Cyc group. However, compared with the Cyc treatments, the EDT treatment decreased the levels of these cytokines (*p* < 0.05 or *p* < 0.01). Cyc administration suppressed the reductions in the levels of IL-1β, IL-17, IL-23 and TNF-α in the EDT group (*p* < 0.05 or *p* < 0.01), as shown in Figure 7(A–D).

### Cyc inhibits the protective effect of EDT against barrier disruption

**Figure 8.**
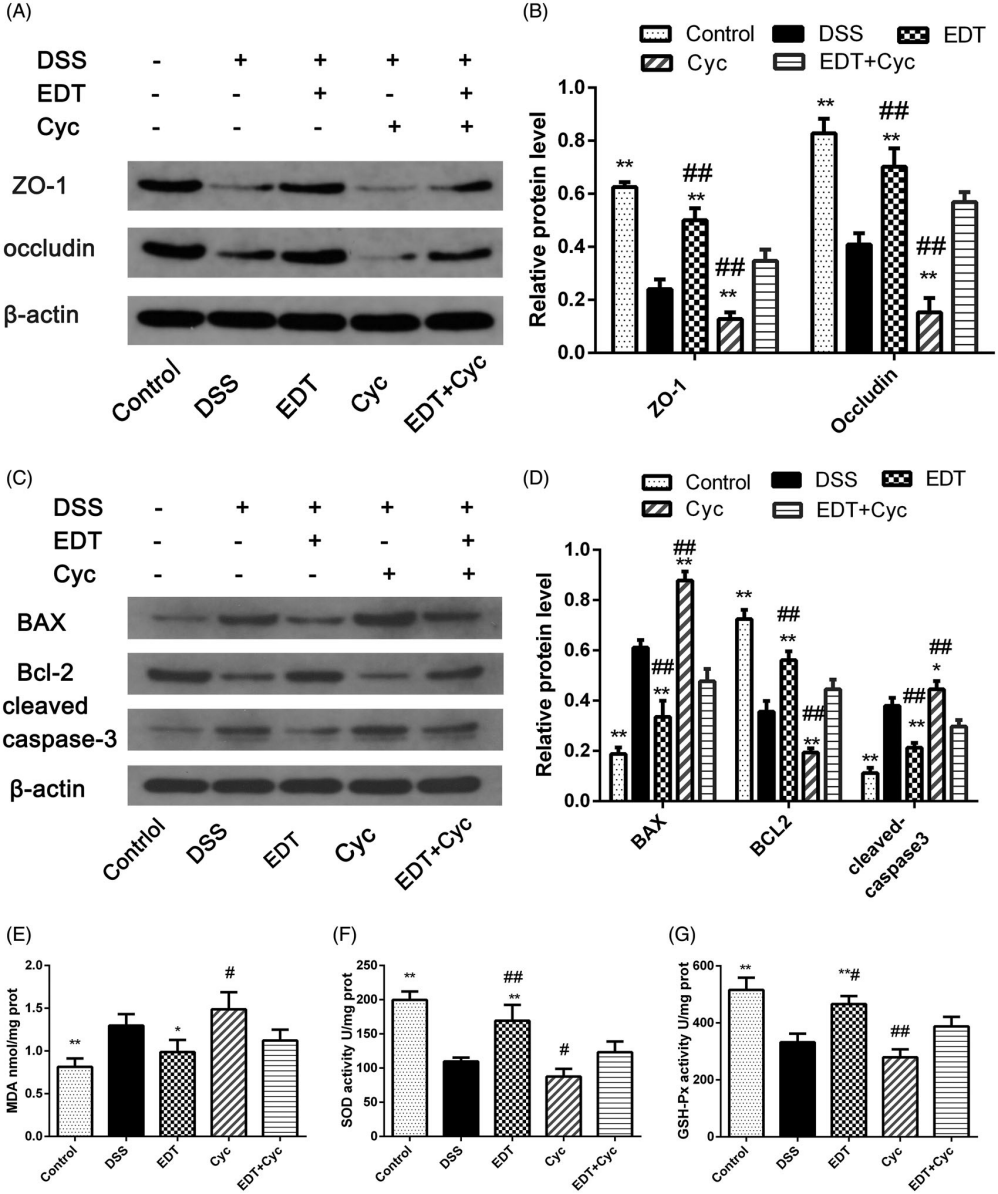
Cyc partially reverses the protective effect of EDT on barrier disruption and its effect on oxidative stress. (A–B) Levels of ZO-1 and occludin were determined using Western blot analyses. (C-D) Levels of Bax, cleaved caspase 3, and Bcl-2 were determined using Western blot analyses. (E) The MDA content, (F) SOD activity, and (G) GSH-Px activity were determined in the colon tissues. Data are presented as means ± S.D. (*n* = 6). DSS: dextran sulphate sodium; EDT: eriodictyol; Cyc: cyclopamine; *p < 0.05 and **p < 0.01 compared with the DSS group. #*p* < 0.05 and ##*p* < 0.01 compared with the EDT + Cyc group.

After Cyc treatment, we analysed the expression levels of ZO-1, occludin, Bax, Bcl-2 and cleaved caspase 3 in mice. As shown in Figure 8(A–D), levels of ZO-1, occludin, and Bcl-2 were further decreased in the Cyc group compared with the control group, and levels of Bax and cleaved caspase 3 were further increased. This result indicates that blocking the Shh pathway promotes intestinal epithelial apoptosis and TJ protein loss. EDT treatment increased levels of ZO-1 (*p* < 0.01), occludin (*p* < 0.01), and Bcl-2 (*p* < 0.05) and decreased those of Bax (*p* < 0.01) and cleaved caspase 3 (*p* < 0.01), which reversed this change. However, the effects of EDT were reduced when mice were treated with EDT and Cyc (*p* < 0.01). This finding suggests that the barrier protection provided by EDT is mediated via the Shh pathway.

### Cyc impairs the effect of EDT on oxidative stress

Mice were treated as described above. Compared with the control group, the DSS group had obviously increased levels of MDA and reduced activity of the antioxidant enzymes SOD and GSH-Px (*p* < 0.05 or *p* < 0.01). After Cyc treatment, the MDA concentration further increased, and the activity of the antioxidant enzymes SOD and GSH-Px further decreased. EDT treatment substantially ameliorated oxidative stress (*p* < 0.01). However, the effect of EDT was reversed by Cyc co-treatment (*p* < 0.05 or *p* < 0.01; Figure 8(E–G)). This result indicates that EDT-mediated attenuation of oxidative stress occurs through the Shh pathway.

### Cyc blocks the activation of the shh pathway

**Figure 9.**
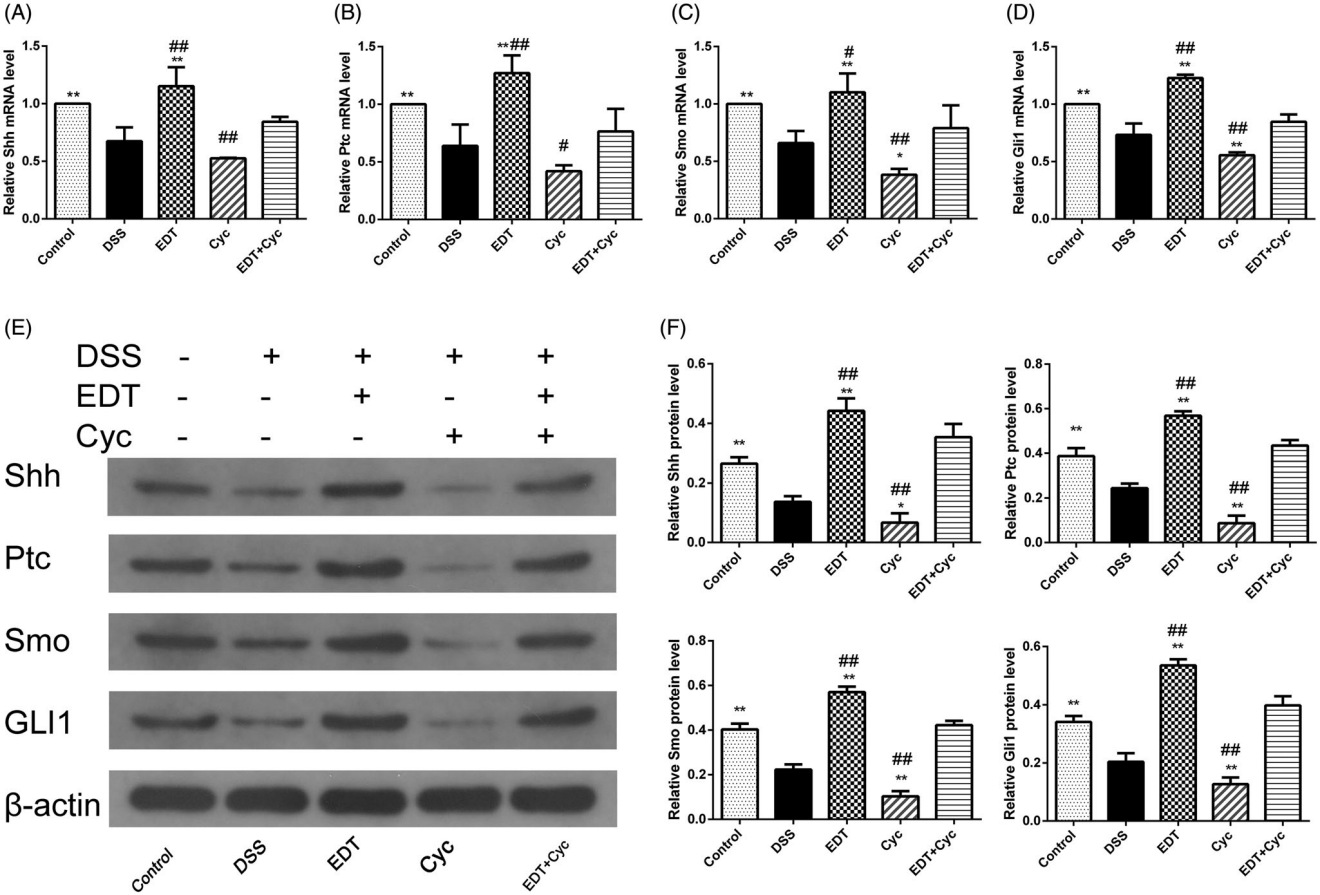
Cyc blocks the activation of the Shh pathway in mice with DSS-induced colitis. (A-D) Levels of the Shh, Ptc, Smo, and Gli1 mRNAs were measured using RT-PCR. (E-F) Levels of the Shh, Ptc, Smo, and Gli1 proteins were determined using Western blot analyses. Data are presented as means ± S.D. (*n* = 6). DSS: dextran sulphate sodium; EDT: eriodictyol; Cyc: cyclopamine. *p < 0.05 and **p < 0.01 compared with the DSS group. #*p* < 0.05 and ##*p* < 0.01 compared with the EDT + Cyc group.

RT-PCR and Western blot analyses showed that the expression of Shh, Ptc, Smo, and Gli1 was significantly increased in the EDT group compared with the DSS model group (*p* < 0.01). However, EDT and Cyc co-treatment also inhibited the increase in the expression level of the Shh pathway components induced by EDT compared to EDT treatment alone (*p* < 0.05 or *p* < 0.01; Figure 9(A–F)).

## Discussion

The therapeutic potential of EDT on many diseases, such as hypercholesterolaemia, colon cancer, cerebral ischaemia, acute kidney injury and neurodegenerative diseases, has attracted increased attention (Jing et al. 2015; Ferreira et al. 2016; Li et al. 2016; Yamashita et al. 2016; Mariyappan et al. 2017). The DSS-induced mouse model of colitis is a well-established experimental model with symptoms and signs similar to those observed in humans with UC, such as weight loss, diarrhoea, wasting, melena, mucosal ulcers, colon shortening, and inflammatory cell infiltration (Chiou et al. 2012). In the present study, we focussed on the protective effects and mechanism of EDT on colitis by establishing a DSS-induced colitis model. First, we found that the DAI scores of the EDT-treated mice were significantly reduced compared with those of the DSS model mice. The length of the colon of the EDT-treated mice was longer than that of the DSS model mice, and each EDT dose significantly reduced the histological score. Therefore, EDT intervention alleviated the severity of experimental colitis in mice.

The inflammatory response and oxidative stress play important roles in the pathogenesis of UC. The activation of pro-inflammatory cytokines, such as TNF-α, IL-1β, and IL-6, in intestinal inflammatory cells can cause a cascade of intestinal inflammation that leads to local inflammation and immune system disorders (Siddique and Khan 2011). In addition to the production of pro-inflammatory cytokines during the immune response, intestinal inflammatory cells can produce ROS, which alters the redox balance in the intestinal mucosa, causing DNA damage, protein oxidation, and lipid peroxidation, further impairing the colonic mucosa and affecting intestinal homeostasis (Bhattacharyya et al. 2014; Pereira et al. 2015). IL 23 and IL-17 are involved in the pathogenesis of IBD. IL-23, a cytokine produced by innate immune cells, such as dendritic cells, macrophages, B cells, or endothelial cells (Sun et al. 2015), is related to local tissue-specific inflammatory responses in the gastrointestinal tract. The level of IL-17, a vital cytokine secreted by Th17 cells, is elevated in IBD patients compared with healthy control individuals (Fujino et al. 2003). IL-23 is closely associated with the differentiation, expansion and stabilisation of Th17 cells. Innate CD4^+^ Th17 cells induce IL-23 receptor expression in response to TGF-β, IL-6, and IL-1β exposure. The interaction of IL-23 with this receptor activates the differentiation of Th17 cells and the secretion of pro-inflammatory factors, including IL-17 (Abraham and Cho 2009). IL-23/IL-17 are involved in the defense against intestinal microbes (Wozniak et al. 2006) and are highly expressed in various IBD models, such as the CD4^+^ CD45RB^hi^ transfer model, IL-10 ^-/-^ colitis model, DSS-induced colitis model and TNBS-induced colitis model; moreover, anti-IL-23 treatment can prevent and treat the symptoms of colitis (Elson et al. 2007). In this study, EDT treatment significantly reduced the levels of TNF-α, IL-1β, IL-23 and IL-17 in colon tissue. We analysed MDA, a commonly used indicator of lipid peroxidation and oxidative stress and found that the MDA concentration was increased in the colon of the DSS group, whereas the activity of antioxidant enzymes (such as SOD and GSH-Px) was significantly reduced. However, EDT treatment reduced the MDA concentration and enhanced SOD and GSH-Px activity. This result reveals that EDT ameliorates the inflammatory response and improves oxidative stress in DSS-induced colitis.

Compromised intestinal barrier integrity is considered to contribute to IBD (Chelakkot et al. 2018). Intestinal epithelial cells (IECs), the mucosal layer and TJ proteins compose the intestinal epithelial barrier. An essential function of IECs is the maintenance of barrier integrity, allowing the permeability of essential ions, nutrients, and water but limiting the entry of bacterial toxins and pathogens (Rescigno 2011). TJ proteins are the most apical epithelial intercellular structures, equipping IECs with this function, which helps to maintain and regulate the epithelial barrier (Diesing et al. 2011). ZO-1 and occludin are crucial components of multiple TJ protein complexes. The loss of TJ proteins is a key factor leading to barrier destruction in models of DSS-induced colitis (Eichele and Kharbanda 2017). IEC apoptosis also disrupts epithelial barrier integrity and further aggravates intestinal inflammation (Shi et al. 2019). IEC apoptosis is increased in patients with UC (Sipos et al. 2002), and Bcl-2, an anti-apoptotic factor, plays a pivotal role in apoptosis. Cleaved caspase 3 and Bax are important pro-apoptotic proteins, and Bax dimerises with Bcl-2 to suppress its effects. In addition, as a transcriptional target, Bcl-2 regulates the Hh pathway and participates in IECs apoptosis (Lees et al. 2005). In this study, we found that occludin and ZO-1 protein expression was obviously upregulated after EDT administration, whereas expression of these proteins in the DSS model group was reduced. Moreover, EDT treatment significantly increased the levels of Bcl-2 and decreased the levels of Bax and cleaved caspase 3. Based on these results, EDT preserves the intestinal epithelial barrier integrity in mice with DSS-induced colitis, consistent with the results of H&E staining.

A large number of studies have shown that the Shh pathway is involved in inflammation, oxidative stress, epithelial barrier function and apoptosis inhibition in various diseases. Activation of the Shh signalling pathway attenuates oxidative stress and hepatocyte apoptosis, promoting hepatocyte regeneration in alcoholic cirrhosis (Wang et al. 2016). In acute pancreatitis, the Shh pathway attenuates inflammation via the upregulation of IL-10 (Zhou et al. 2012). Recombinant human Shh protein has been reported to upregulate ZO-1 and occludin expression to repair damaged TJs between cerebral endothelial cells, thereby alleviating blood-brain barrier leakage after ischaemic stroke in rats (Xia et al. 2013). In another study, gastric epithelial cell–cell junctions were disrupted in conditional Shh knockout mice (Xiao et al. 2010). Moreover, Hh signalling has consistently been shown to be involved in DSS-induced colitis and colitis-associated colon cancer (Lees et al. 2008; Zacharias et al. 2010; Gerling et al. 2016; Lee et al. 2016). In another recent study, pharmacological activation of the Shh pathway significantly reduced oxidative stress and apoptosis in a model of DSS-induced colitis by regulating MDA, SOD, Bax/Bcl-2 and caspase 3 levels, and Cyc treatment abrogated the protective effect (Lv et al. 2018). In this study, we found that the Shh pathway was markedly activated by EDT via increased expression of Smo, Ptc, Shh and Gli1. Next, the Cyc, a specific Smo receptor inhibitor, was used to verify whether Shh pathway activation is involved in the protective effect of EDT against DSS-induced colitis. We found that after the administration of Cyc, the downregulated expression of inflammatory cytokines (IL-1β, IL-17, IL-23 and TNF-α) was reversed and that oxidative stress and epithelial barrier disruption were further aggravated. These results indicate that activation of the Shh pathway is involved in EDT-mediated protection against DSS-induced colitis.

This study was only a preliminary analysis of the basic role of EDT in colitis mice in which a possible relationship between EDT and the Shh pathway was explored. Although DSS-induced colitis mice can be effectively treated with certain concentrations of EDT, it remains unclear whether the drug is safe for human use. In addition, treatment with an Shh pathway inhibitor partially reversed the effect of EDT, but did not completely eliminate the effect, indicating that the targets of EDT may be diverse and that Shh signalling may be one of the pathways through which EDT protects against colitis. Therefore, additional experimental studies are needed to translate these results for clinical applications in UC patients. Further studies on other protective mechanisms of EDT against colitis and the effect of EDT on UC patients should be conducted to evaluate the clinical application of EDT.

## Conclusions

This study demonstrated the protective effects of EDT on DSS-induced colitis and showed that the Shh pathway can upregulate expression of the TJ proteins ZO-1 and occludin, reducing colitis-induced epithelial barrier damage. In summary, we deduced that EDT inhibits the inflammatory response, oxidative stress and epithelial barrier disruption in the colon by activating the Shh pathway. These results indicate that EDT might be a potential treatment for UC.
